# Vitamin C: A Review on its Role in the Management of Metabolic Syndrome

**DOI:** 10.7150/ijms.47103

**Published:** 2020-06-27

**Authors:** Sok Kuan Wong, Kok-Yong Chin, Soelaiman Ima-Nirwana

**Affiliations:** Department of Pharmacology, Faculty of Medicine, Universiti Kebangsaan Malaysia, Jalan Yaacob Latif, Bandar Tun Razak, 56000 Cheras, Kuala Lumpur, Malaysia.

**Keywords:** antioxidants, ascorbic acid, ascorbate, inflammation, oxidative stress.

## Abstract

Oxidative stress and inflammation are two interlinked events that exist simultaneously in metabolic syndrome (MetS) and its related complications. These pathophysiological processes can be easily triggered by each other. This review summarizes the current evidence from animal and human studies on the effects of vitamin C in managing MetS. *In vivo* studies showed promising effects of vitamin C, but most of the interventions used were in combination with other compounds. The direct effects of vitamin C remain to be elucidated. In humans, the current state of evidence revealed that lower vitamin C intake and circulating concentration were found in MetS subjects. A negative relationship was observed between vitamin C intake / concentration and the risk of MetS. Oral supplementation of vitamin C also improved MetS conditions. It has been postulated that the positive outcomes of vitamin C may be in part mediated through its anti-oxidative and anti-inflammatory properties. These observations suggest the importance of MetS patients to have an adequate intake of vitamin C through food, beverages or supplements in order to maintain its concentration in the systemic circulation and potentially reverse MetS.

## Introduction

The harmonised criteria according to Joint Interim Statement define metabolic syndrome (MetS) in the presence of at least three out of these five metabolic abnormalities: elevated waist circumference, blood pressure (BP), fasting blood glucose (FBG), triglycerides (TG) and reduced high-density lipoprotein cholesterol (HDL-C) 1. The unhealthy dietary pattern, such as high consumption of refined carbohydrate and saturated fat, is directly correlated with MetS development 2-5. The lack of moderate-to-vigorous physical activity is also one of the important risk factors for MetS 6. MetS patients are advised to adopt lifestyle-based interventions as the initial management, such as avoiding food and beverage containing high sugar and fat content along with maintaining aerobic exercise. Drug therapies (medications for obesity, hyperglycaemia, hypertension and hypercholesterolemia) and bariatric surgery are recommended if lifestyle modifications are not successful in improving MetS conditions.

Recently, there is a growing interest in employing anti-oxidative and anti-inflammatory agents as prophylactic or therapeutic agents against MetS 7-10, in line with the speculation that oxidative stress and inflammation play a significant role in the pathophysiology of MetS. Under this hypothesis, oxidative damage and inflammation are triggered by exogenous factors, like an overabundance of dietary carbohydrate and lipid. Dietary fat mainly consists of a combination of saturated fatty acid (SFA), trans-unsaturated fatty acid (TFA), monounsaturated fatty acid (MUFA) and polyunsaturated fatty acid (PUFA) with different health effects 11. The degree of saturation in dietary fat has been proposed to differentially influence the several key factors of MetS. Both SFA and TFA are highly atherogenic by increasing the levels of total cholesterol (TC), low-density lipoprotein cholesterol (LDL-C), very low-density lipoprotein cholesterol (VLDL-C), apolipoprotein A-1 (apoA1) and decreasing the level of HDL-C. In contrast, MUFA and PUFA have favourable effects on atherogenicity by reducing TC, LDL-C and elevating HDL-C 11. Excessive macronutrients ingestion generates a large amount of reactive oxygen species (ROS) as by-product causing lipid peroxidation and oxidative stress that eventually lead to inflammation via activation of nuclear factor-kappa B (NF-κB) signalling pathway 12,13. Likewise, the increase in adipocyte size and number arise from abdominal obesity and dyslipidaemia elevate the endogenous pool of pro-inflammatory cytokines, which act as potent stimulators for ROS production by macrophages and monocytes 14. Therefore, the interdependence of oxidative stress and inflammation suggests the potential use of agents exhibiting anti-oxidative and anti-inflammatory activities simultaneously to mitigate MetS.

Vitamin C, known as ascorbic acid or ascorbate, is an essential water-soluble micronutrient traditionally used to prevent and treat scurvy. Vitamin C is present universally in both plants and animals. The major dietary sources of vitamin C are fresh fruits and vegetables. Vitamin C has been suggested to be beneficial in reversing MetS-associated abnormalities based on several considerations. Plasma vitamin C concentration was inversely associated with body mass index (BMI), percentage of body fat and waist circumference 15. Vitamin C supplementation resulted in significant decreases in blood glucose 16, BP 17, TG and LDL-C 18. Moreover, vitamin C is a powerful antioxidant because it acts as a reducing agent preventing other compounds from being oxidised. By donating electrons, vitamin C scavenges harmful free radicals leaving the ascorbyl radical, which is relatively stable and unreactive 19. Previous reports have confirmed the ability of vitamin C in reducing oxidative stress 19,20. Vitamin C also resolves the inflammatory response by influencing neutrophil chemotaxis in response to inflammatory mediators, enhancing phagocytosis of microbes by neutrophils and supporting neutrophil clearance by macrophages 21.

This review summarizes the current knowledge on the effects of vitamin C in combating MetS in animal and human studies. The anti-oxidative and anti-inflammatory properties of vitamin C as the primary mechanisms regulating the physiopathology of MetS are also discussed. This review may be instructive for people with MetS, healthcare professionals who care for individuals with MetS and researchers who conduct studies on MetS.

### Evidence acquisition

**Figure 1:**
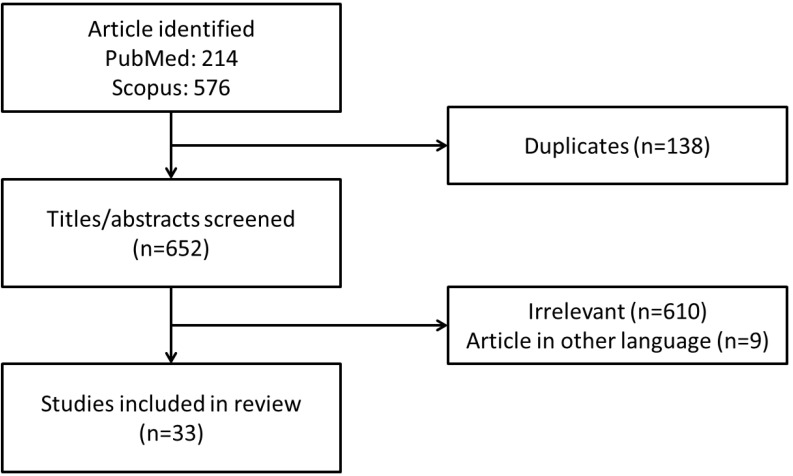
Framework for the selection of relevant studies.

A literature search was conducted using PubMed and Scopus databases from February 15, 2020 to March 15, 2020 with keywords “(vitamin C OR ascorbic acid OR ascorbate) AND metabolic syndrome”. Our literature search identified 214 records from PubMed and 576 records from Scopus. After removing duplicated articles (n=138), the titles and abstracts were screened to remove irrelevant articles (n=610) and articles in other languages (n=9). Only articles written in English and Mandarin were included. In this review, we focus on summarising the effects of vitamin C on MetS as an entity in animals and humans. The use of vitamin C as monotherapy and/or combined therapy were included. Articles with the main purpose of investigating (a) the association between vitamin C intake or its circulating concentration in blood and risk of MetS as well as (b) the effects of vitamin C supplementation on MetS as the primary outcomes were retrieved. A total of 33 articles which met the criteria were included in this review (Figure 1).

### The effects of vitamin C on MetS: evidence from animal studies

The effects of vitamin C supplementation on MetS have been explored in rodents and rabbits (Table 1). Bilbis et al. (2012) investigated the effects of vitamin C in the management of MetS traits using hypertensive Wistar rats placed on high salt [8% sodium chloride (NaCl)] diet. Supplementation of vitamin C for 4 weeks decreased the percentage weight gain, systolic BP, glucose, insulin, insulin resistance [evidenced by decreased homeostatic model assessment of insulin resistance (HOMA-IR)], TC, TG, LDL-C, VLDL-C, atherogenic index and increased vitamin C concentration in the salt-loaded animals 22. However, salt-induced hypertension is not a good model of MetS as high dietary salt is not the only factor contributing to the occurrence and progression of MetS in humans. Thus, this animal model does not resemble the human disease state of MetS. In a different animal model, Lebel et al. (2010) utilised a Werner syndrome mouse model to examine the effects of sodium ascorbate (0.4% w/v) on metabolic abnormalities. Werner syndrome is an autosomal recessive genetic disorder, characterized by accelerated ageing and displaying features of skin atrophy, wrinkles, loss of hair, atherosclerosis, as well as abnormal glucose and lipid metabolism 23. The outcomes of the study indicated that ascorbate-supplemented drinking water decreased visceral fat weight, TG, blood glucose and normalised insulin resistance index (evidenced by decreased HOMA-IR) in liver and heart of mutant Wrn*^∆hel/∆hel^* mice (a knockout mouse model generated by deleting the RecQ helicase domain of the mouse Wrn homologue gene) 24. In diabetic rabbits induced by alloxan monohydrate, administration of vitamin C (150 mg/kg) dissolved in drinking water for 2 weeks reversed the elevated mean blood glucose, systolic and diastolic BP and TG level. The concentration of HDL-C in the diabetic rabbits administered with vitamin C was increased as compared to the non-treated controls 25.

Researchers have also attempted to elucidate the effects of vitamin C in combination with other antioxidants, such as polyphenols or vitamins in managing MetS. Two studies were conducted by the same group of researchers to study the impact of an antioxidant cocktail containing *S*-adenosylmethione (0.5 g/kg diet), vitamin C (12.5 g/kg diet) and vitamin E (1.5 g/kg diet) in the development of hepatic insulin sensitizing substance (HISS)-dependent insulin resistance (HDIR) and adiposity with increasing age 26,27. In the first study, male Sprague-Dawley rats were divided into two groups: one group received standard chow diet while the other group received standard chow diet enriched with an antioxidant cocktail. At the age of 12 months, the intake of antioxidant cocktail resulted in decreases in total fat pad mass, perienteric fat mass and epididymal fat mass as compared to the rats of the same age but without antioxidants in the diet. Besides, ageing rats receiving antioxidant cocktail from the diet showed improved HDIR as shown by higher rapid insulin sensitivity test (RIST) index and HISS component than the group not receiving the antioxidant cocktail 26. In the subsequent year, male Wistar rats were assigned with a standard diet supplemented with an antioxidant cocktail and 5% sucrose drinking water. Comparing to the age-matched non-treated sucrose-fed animals, the administration of antioxidant cocktail via diet blunted the elevation of fasting glucose, postprandial glucose, fasting insulin, postprandial insulin, whole body fat mass, visceral fat mass, TG but increased HDL-C. Similar to the previous study, improved insulin sensitivity was noted in the sucrose-fed animals treated with an antioxidant cocktail 27.

Using high salt diet-induced hypertensive rats, treatment with combined vitamins (6 mg/kg of vitamin A, 100 mg/kg of vitamin C and 60 mg/kg of vitamin E) prevented MetS component (indicated by reductions in weight gain, BP, glucose level, insulin resistance and improved lipid profile) compared to the non-treated hypertensive rats 22. Recently, Gao et al. (2015) tested a fixed-dose combination of natural antioxidants consisting vitamin C, green tea polyphenol and grape seed extract proanthocyanidin on MetS using male Sprague-Dawley rats and female type 2 diabetes mellitus (T2DM) KK-ay mice fed on a high-fat diet. Their data revealed that the combination of antioxidant decreased body weight, average fat coefficient, average liver coefficient, total amount of fat in epididymal and perirenal white adipose tissue, size of cell in adipose tissue, TG, LDL-C, FBG, random blood glucose (RBG), 2-hour postprandial blood glucose (PBG) and increased HDL-C in high-fat diet-induced MetS rats. In KK-ay mouse model treated with a combination of antioxidants, the diabetic phenotype and lipid disorders were ameliorated (characterized by lowered FBG, RBG, PBG, TC, TG, LDL-C and raised HDL-C) 28.

Among the animal studies included in this review, some studies investigated the effects of vitamin C alone, while some studies evaluated the integrative effects of vitamin C and other compounds. Even though findings from these studies showed promising effects of vitamin C supplementation in combating MetS, further validations are necessary owing to the limited animal studies on the effects of vitamin C alone. It is rather challenging to conclude that vitamin C is responsible for the observed beneficial effects when the animals were treated with the combination of vitamin C with other compounds. The comparison between study outcomes is also difficult to performed due to the heterogeneity of compounds as the intervention. Another important aspect to note is the ability to compensate for vitamin C through L-gulonolactone in animals. Thus, the consumption of diet containing vitamin C may not change the plasma concentration of vitamin C. In this case, the use of animals is not an ideal model to mimic human situation. Study should be carried out to measure and compare plasma vitamin C concentration before and after supplementation of vitamin C. The positive outcomes from animal studies also prompt the need to look further into the available evidence on the association between vitamin C and MetS in humans.

### The effects of vitamin C on MetS: evidence from human studies

In this section, the potential of vitamin C in reversing MetS in human as evidenced by cross-sectional and case-controlled studies will be summarized. The current documented literature showed heterogeneous findings, whereby possible or negligible relationships between vitamin C intake / concentration and MetS have been reported. The interventional trials conducted by researchers also revealed positive or no effect of vitamin C supplementation on MetS conditions (Table 2).

#### The relationship between vitamin C intake and MetS

Three relatively large cross-sectional studies were conducted among the general Korean population to investigate the association between vitamin C intake and risk for MetS. All these studies used data from the Korea National Health and Nutrition Examination Survey (KNHANES). Between the year 2007 until 2012, daily intake of vitamin and MetS parameters were collected among 27,656 adults with ≥ 20 years of age. The authors found that the vitamin C intake was lower in the MetS group (73.4 ± 1.2% of the recommended intake) than non-MetS group (80.1 ± 0.7% of the recommended intake). With a two-fold increase in total vitamin C intake in women, the incidence of MetS decreased [odd ratio (OR) = 0.933; 95% confident interval (CI) 0.883 to 0.987] 29. Kim & Choi (2016) analysed information from 22,671 adults aged 20 years or older to examine the integrative effects of physical activity and dietary vitamin C on the risk of MetS. They found a substantial decrease in waist circumference in individuals with high vitamin C intake alone (OR = -0.3; 95% CI -0.6 to -0.1), high physical activity alone (OR = -0.6; 95% CI -0.8 to -0.4) as well as both high vitamin C intake and physical activity (OR = -1.0; 95% CI -1.2 to -0.8). Apart from that, TG level was decreased whereas HDL-C level was increased in individuals with both high vitamin C intake and physical activity. Consistent findings were obtained after sub-analysis based on sex was performed. They also found lower risk of MetS in the high vitamin C intake alone (OR = 0.89; 95% CI 0.80 to 0.99), high physical activity alone (OR = 0.81; 95% CI 0.73 to 0.90) as well as both high vitamin C intake and physical activity groups (OR = 0.79; 95% CI 0.71 to 0.87) 30. Another study was conducted using data between 2013 until 2016 composed of 10,351 adults aged 19 - 64 years. In men, the prevalence of MetS was lower in the highest tertile of vitamin C intake than the lowest tertile (OR = 0.75; 95% CI 0.58 to 0.95). In women, TG level was lower in the highest tertile of vitamin C intake compared to the lowest tertile (OR = 0.75; 95% CI 0.61 to 0.93) 31.

In line with these studies, Li and co-researchers reported that MetS patients (n=221; aged 54.2 ± 5.73 years) exhibited lower vitamin C intake than control subjects (n=329; aged 53.3 ± 5.83 years) after adjusting for confounding factors, such as age and sex 32. A reverse correlation was found between vitamin C intake and risk of MetS in three different study populations, i.e. patients with colorectal cancer (OR = 0.89; 95% CI 0.84 to 0.94) 33, volunteers undergoing regular health check-up at Xiangya Hospital Health Management Centre in China (OR = 0.64; 95% CI 0.43 to 0.94) 34 and Saudi adults (OR = 4.1; 95% CI 1.6 to 8.3) 35. Other observations found in these studies were the significantly higher consumption of vitamin C in colorectal cancer patients without MetS (92.7 ± 21.3 mg) than those with MetS (63.1 ± 34.0 mg) 33 and vitamin C intake displayed a negative association with waist circumference 34. Godala and colleagues examined the Polish population consisting of 90 clinically healthy women (aged 57.48 ± 5.79 years) and 184 women with MetS (aged 57.38 ± 8.17 years) and reported that the optimal level of 90 - 110% for vitamin C intake was only achieved in 8.88% of women with MetS which was significantly less often than in the control group 36.

A report recently published by Agarwal et al. (2019) provided an updated evaluation of the association between 100% fruit juice consumption with nutrient intake and risk factors for MetS 37. The study population in this study was 10,112 adults aged 19 years old and above participating in NHANES 2013 - 2016. The consumers of 100% fruit juice (defined as those consuming any amount of 100% fruit juice during the first 24 hours dietary recall) had a significantly higher intake of vitamin C compared to non-consumers, with the intake of vitamin C increased as the 100% fruit juice consumption level increased. As compared to the non-consumers, the 100% fruit juice adult consumers had lower BMI, body weight, waist circumference, plasma glucose and glycated haemoglobin (HbA1c). The risk for being overweight or obese (OR = 0.78; 95% CI 0.65 to 0.95), raised waist circumference (OR = 0.69; 95% CI 0.56 to 0.85) and having MetS (OR = 0.73; 95% CI 0.58 to 0.93) was also decreased in adult consumers of 100% fruit juice 37.

Godala et al. (2016b) did not find a correlation between vitamin C intake from the diet and the plasma concentration of vitamin C in MetS patients recruited from the Department of Internal Medicine and Nephrodiabetology, Medical University of Lodz, Poland. The authors postulated that the increased oxidative stress in the MetS group was counteracted by greater consumption of vitamin C in neutralising ROS, thus decreased concentration of plasma vitamin C could be expected 38. A study by Ford et al. (2003) also reported no difference in dietary intake of vitamin C among participants involved in NHANES 1988 - 1994 with and without MetS (n=8,808; aged ≥20 years). This observation might have resulted from similar vitamin or mineral use during the previous 24 hours and past month 39. These studies found two major outcomes on vitamin C intake in MetS subjects: (a) there was no correlation between vitamin C intake and plasma concentration of vitamin C and (b) there was no difference between dietary intake between the subjects with and without MetS. As compared to the previously aforementioned studies, the association between vitamin C intake and the prevalence of MetS was not evaluated in these two studies. Owing to the vitamin C intake data in these studies served as an estimation and it can be prone to a number of confounders, the vitamin C status in plasma or serum would provide a more accurate representation.

#### The relationship between circulating vitamin C concentration and MetS

Four cross-sectional studies involving adolescents and adults participating in National Health and Nutrition Examination Survey (NHANES) demonstrated that serum vitamin C concentration was inversely correlated with MetS outcomes 39-42. Using data from NHANES between 1988 until 1994, the serum vitamin C concentration was compared among 8,808 adults (aged 20 years and above) with and without MetS. Findings from this study indicated that the age-adjusted concentration of vitamin C was lower in participants with MetS (36.41 ± 1.11 mmol/L) than those without MetS (42.94 ± 0.83 mmol/L). The concentration of vitamin C was inversely correlated with waist circumference (β = -4.090 ± 0.860) and hyperglycaemia (β = -3.066 ± 1.017). Besides, individuals with the highest number of MetS criteria had the lowest serum vitamin C concentration 39. From the year 2001 to 2006, Beydoun and colleagues performed two cross-sectional studies recruiting 4,285 adolescents (aged 12 - 19 years) and 1,574 adults (aged 20 - 85 years), respectively. In adolescents, lower concentration of vitamin C in serum was noted in participants with MetS than those without MetS. Inverse relationships were constantly found between vitamin C and MetS using different models, i.e. after controlling for socio-demographic and dietary factors (OR = 0.26; 95% CI 0.11 to 0.62) as well as after inclusion of serum cholesterol and TG (OR = 0.16; 95% CI 0.05 to 0.52) 40. A similar trend was detected in adults whereby MetS subjects displayed lower vitamin C concentration compared to non-MetS subjects. Vitamin C was inversely related to MetS count and binary outcomes. Every increase of ~28.5 μmol/L in vitamin C concentration was associated with 24% lower prevalence odds of MetS (OR = 0.76; 95% CI 0.58 to 0.98) 41. More recently, a cross-sectional study was published consisting of data from NHANES between 2005 until 2006. A total of 2,049 MetS subjects were identified using the harmonized criteria from the Joint Interim Statement in this study. The authors unveiled that vitamin C concentration decreased with increasing BMI and number of MetS components. In addition, having lower than the clinical reference range for vitamin C was significantly associated with a greater likelihood of MetS (OR = 1.39; 95% CI 1.01 to 1.90) 42.

Another cross-sectional study by Chielle et al. (2018) recruited 85 Brazilian adults (36 MetS subjects and 48 healthy volunteers) aged 22 - 85 years from January to May 2017. The MetS group had a significant decrease in vitamin C concentration when compared to the group without MetS 43. Comparative results were obtained from case-control studies assessing the relationship between plasma vitamin C status and MetS components. Odum and co-researchers recruited 200 Nigerians consisting of 100 MetS subjects as well as 100 age- and sex-matched controls. The mean plasma vitamin C concentration of the study population was measured and a significantly lower concentration of vitamin C was observed in MetS individuals as compared to the healthy subjects 44,45. Two case-control studies by Godala and co-researchers estimated the plasma vitamin C concentration in MetS patients (aged 30 - 65 years) and healthy subjects (aged 41 - 65 years). MetS subjects were found to have a lower plasma vitamin C concentration relative to healthy controls. Deficiency of circulating vitamin C concentration was significantly more common in MetS subjects than healthy individuals. The concentration of vitamin C in MetS patients was found to inversely correlate with systolic BP (Pearson's coefficient: -0.31, p<0.0001), diastolic BP (Pearson's coefficient: -0.1689, p=0.042) and HDL-C (Pearson's coefficient: -0.19, p=0.018) 38,46.

On the contrary, two cross-sectional studies reported paradoxical results. A total of 118 healthy participants working at B.P. Koirala Institute of Health Sciences, Dharan, Nepal were included in a cross-sectional study. They reported that 39% subjects (n=46) were diagnosed with MetS and no significant difference was observed in the plasma vitamin C concentration among individuals with and without MetS. Furthermore, other oxidative stress parameter and antioxidant levels including malondialdehyde (MDA), reduced glutathione (GSH), glutathione peroxidase (GPx), superoxide dismutase (SOD) and vitamin E were not significantly different among the subjects with and without MetS. The authors suggested that oxidative stress did not contribute in the pathogenesis of MetS in this population 47. Wang et al. (2019) conducted a cross-sectional study among 205 Chinese women consisting of 65 healthy women, 150 women with polycystic ovary syndrome (PCOS) as well as 55 women with PCOS and MetS (aged 21 - 40 years). Findings from this study demonstrated no difference in serum vitamin C concentration among the three groups of subjects. The discrepancy observed in the findings of this study compared to other studies might be due to the presence of other antioxidants in counteracting the oxidative stress during MetS. The levels of SOD and total antioxidant activity were lower in PCOS women with MetS as compared to healthy control 48.

#### The effects of vitamin C supplementation on MetS

The earliest clinical trial investigating the effects of vitamin C on MetS was conducted by Czernichow et al. (2009). They designed a randomised double-blind placebo-controlled, primary prevention trial, known as the French SUpplementation en VItamines et Mineraux AntioXydants (SU.VI.MAX) study. A total of 5,220 men and women were included and randomly assigned to receive either a supplement containing a combination of antioxidants (120 mg vitamin C, 30 mg vitamin E, 6 mg β-carotene, 20 mg zinc and 100 μg selenium) or a placebo. The participants were free of MetS during the onset of the study and were followed for 7.5 years. Results from this study pinpointed that antioxidant supplementation for 7.5 years did not affect the incident risk of MetS, as shown by an approximately equal number of MetS events in the placebo and intervention groups. However, higher baseline serum vitamin C concentration was associated with lower odds for MetS incident risk (OR = 0.53; 95% CI 0.35 to 0.80) 49.

The effects of vitamin C supplementation alone or in combination with endurance physical activity were investigated by Farag and co-authors in two randomised controlled trials. In the first study, participants of both sexes with MetS (n=141; aged 30 - 50 years old) were randomly divided into six groups receiving placebo, 2000 IU/day vitamin D or 500 mg/day vitamin C with or without 30 minutes/day physical activities for three months. Their data concluded that subjects taking vitamin C alone had a lowered TG level compared to placebo. The level of HDL-C was increased, but TG and waist circumference were decreased in MetS patients subjected to vitamin C supplementation with exercise in relative to the placebo 50. Another randomised double-blind, placebo-controlled trial by the same group of researchers involved 96 MetS patients aged between 30 to 60 years old. It was worthy to note that vitamin C supplementation alone decreased BMI and LDL-C compared to the placebo group. The MetS patients had lower systolic BP after taking vitamin C supplements and undergoing physical activity for three months than the placebo controls 51.

Recently, Mahmoodi et al. (2019) designed a double-blind controlled trial to elucidate the influence of zinc (5 mg) and vitamin C (300 mg) in combination on MetS parameters in postmenopausal women with T2DM (n=69; aged 50 - 65 years). The supplementation of zinc plus vitamin C for 12 weeks resulted in higher FBG and HDL-C as well as lower systolic and diastolic BP 52. In the same year, Ponce et al. (2019) tested whether adopting a balanced diet alone or adopting a balanced diet plus daily intake of orange juice attenuated risk factors in individuals with MetS (n=72; aged 48 ± 9 years). Subjects who were consuming a balanced diet and orange juice daily (328 ± 35 mg) obtained a significantly higher concentration of vitamin C in their nutritional composition of the meal as compared to those who only took a balanced diet (145 ± 42 mg). Both interventions improved MetS features, indicated by decreased body weight, BMI, waist circumference, fat mass, visceral fat area, blood glucose, TC, HDL-C and BP. Only the combination of a balanced diet together with the intake of orange juice lowered insulin and insulin resistance 53. Shenoy et al. (2010) also evaluated the impact of a ready serve vegetable juice on MetS-associated parameters among men and women (n=81; aged 35 - 65 years) who met the criteria for MetS set by National Cholesterol Education Program (NCEP) Adult Treatment Panel (ATP) Panel III. The participants were randomised into three groups receiving no vegetable juice, 8 or 16 ounces of low sodium vegetable juice per day for 12 weeks. The authors pointed out that the groups provided with vegetable juice had a higher intake of vitamin C and lost more weight as compared to the group who did not drink the juice 54.

The current evidence shows that high vitamin C intake, concentration and supplementation are beneficial in reversing MetS except for a few studies. Several key points can be summarized based on the findings from the aforementioned studies. Firstly, MetS subjects had lower vitamin C intake and circulating concentration. Secondly, low vitamin C intake and circulating concentration are closely linked with a high risk of MetS. Thirdly, there was no correlation between vitamin C consumption and plasma concentration. Hence, it has been postulated that vitamin C was consumed when neutralizing inflammation and free radicals. Greater consumption of vitamin C was needed due to higher inflammatory response and oxidative stress in people with MetS. Furthermore, all randomised controlled trial included in this review investigated the effects of vitamin C supplementation in combination with other antioxidants or exercise on MetS. Similar to animal studies, it is hard to justify whether the beneficial effects in improving MetS abnormalities were contributed by vitamin C or other compounds that present in the combined therapy. However, it can be suggested that the supplementation of vitamin C in combination with other antioxidants or exercise may provide synergistic effects in the management of MetS and its associated conditions.

### The potential mechanism of action for vitamin C in the management of MetS

#### The anti-oxidative property of vitamin C

Oxidative stress, an imbalance between the production and inactivation of ROS, is the hallmark of MetS 55. Several biochemical mechanisms of ROS formation during MetS have been postulated. Unhealthy eating habits (such as consuming a diet rich in fat and carbohydrate), as well as low physical activity, are the contributing factors of MetS 6,56. In the state of overnutrition, the large flux of macronutrients exacerbates oxidation process resulting in higher ROS generation and postprandial oxidative stress response 57. The increase in adipose tissues stimulates excessive production of pro-inflammatory mediators, which in turn stimulate macrophages and monocytes to generate ROS 14. Hypertrophied adipocytes also secrete angiotensin II to enhance ROS production from nicotinamide adenine dinucleotide phosphate (NADPH) oxidase 58. Under physiological condition, the synthesis of ROS is often counteracted by the natural antioxidant system in the body consisting of a series of enzymatic and non-enzymatic antioxidants. The examples of enzymatic antioxidants are SOD, GPx and catalase (CAT) whereas the non-enzymatic antioxidants include GSH, vitamin C, vitamin E, beta (β)-carotene and other phytochemicals. The perturbation of ROS and antioxidant balance is often due to an increase in ROS production or/and a depression of the antioxidant system. High level of ROS reacts with cellular macromolecules and causes lipid peroxidation 59. Thus, the products of lipid peroxidations are often the biomarkers for oxidative stress.

The protective effects of vitamin C against oxidative stress have been presented in MetS animal models (Table 1). Using Wistar rats fed with a high-salt diet, the orally supplemented 100 mg/kg vitamin C for 4 weeks increased vitamin C concentration, total antioxidant status and decreased MDA content in animals as compared to non-treated negative controls 22. Another study showed that the mutant Wrn*^∆hel/∆hel^* mice treated with drinking water enriched with 0.4% sodium ascorbate decreased oxidative stress in liver and heart tissues [the levels of ROS and deoxyribonucleic acid (DNA) damage decreased] along with the reversal of metabolic phenotypes 24. In the rabbit model, results from comet assay showed that the DNA damage in lymphocytes of the diabetic group without supplementation of vitamin C was higher (indicated by longer tail length) and a reduction was seen in the vitamin C-treated group. Simultaneously, serum MDA was also higher in diabetic group and decreased after vitamin C supplementation. The serum paraoxonase-1 (PON-1) activity and time required for small dense low-density lipoprotein (sdLDL) oxidation were low in the diabetic group. These parameters increased after vitamin C supplementation 25. The investigation using animal model of MetS induced by high-fat diet displayed MetS features and high degree of oxidation (MDA level) in the body. Feeding the animals with a fixed-dose combination of vitamin C, green tea polyphenols and grape seed extract proanthocyanidin successfully ameliorated the oxidative stress 28.

In a cross-sectional study, Li et al. (2013) found that MDA content was significantly higher, whereas the SOD activity and β-carotene level were significantly lower in the MetS patients. Serum SOD and GPx activity were decreased as the number of MetS components increased. Higher SOD activity (OR = 0.506; 95% CI 0.303 to 0.844) and β-carotene level (OR = 0.097; 95% CI 0.026 to 0.374) were associated with lower odds of MetS after adjusted for age and gender. The analysis of correlation found a positive association between vitamin C intake and serum antioxidant level 32. MetS parameters and total antioxidant capacity adjusted by daily energy intake were measured by Puchau et al. (2010) in a study involving 153 Caucasian healthy young subjects (aged 20.8 ± 2.7 years). The results showed that dietary total antioxidant capacity was positively associated with vitamin C (r = 0.29, p < 0.001) but negatively associated with body weight (r = -0.18, p = 0.025), waist circumference (r = -0.18, p = 0.029), waist-to-hip ratio (r = -0.16, p = 0.048), systolic BP (r = -0.19, p = 0.018) and serum glucose (r = -0.26, p = 0.001). Serum free fatty acid was also appeared to be negatively associated with dietary total antioxidant capacity in multiple linear regression analysis (OR = -0.09; 95% CI -0.16 to -0.03) 60. In line with these studies, it was observed that the level of γ-glutamyl transferase (GGT) was increased as the number of MetS indicators increased among the population in the United States indicating damage to the liver and bile ducts 42. Several oxidative markers were evaluated in Brazilian adult with and without MetS. The MetS subjects presented the increases in GGT, glutamic-pyruvic transaminase (GPT), glutamic-oxaloacetic transaminase (GOT), total ferric antioxidant power (FRAP), sulfhydryl groups (SH) and thiobarbituric acid reactive substances (TBARS) 43. By contrasting the oxidative stress indicators and anti-oxidative capacity in healthy controls, the MDA level was higher whereas the levels of SOD and total antioxidant activity were lower in PCOS subjects with MetS 48. In a randomised controlled trial, MetS patients assigned with a balanced diet and 500 mL/day orange juice over 12 weeks had higher vitamin C intake with a concomitant increase in antioxidant capacity 53.

#### The anti-inflammatory property of vitamin C

Chronic low-grade inflammation is a common feature accompanying MetS and its associated complications 61. It is characterized by the activation of inflammatory signalling networks resulting in dysregulation of adipokines, overwhelming production of cytokines and chemokines in the systemic circulation 62. Generally, the hyperplasia and hypertrophy of adipose tissue (the main source of various inflammatory mediators) contribute to the development of MetS-associated inflammation. High levels of inflammatory mediators further promote the recruitment and accumulation of macrophages in adipose tissue, exacerbating the state of inflammation during MetS 63. Humans with MetS experience chronic systemic inflammation. In addition, adiposity is also asscociated with increased level of inflammatory markers, indicating that adipose tissue is a significant contributor of inflammatory conditions in MetS 64. Obesity, in the absence of MetS, is also known to be associated with an inflammatory state. The presence of other MetS features (hyperglycaemia, hypertension and dyslipidaemia) may exacerbate the inflammatory condition because each component of MetS brings about the increase in local and systemic production of pro-inflammatory cytokines 65-67. A human study by Genel et al. (2014) indicated that inflammation was more prominent in MetS subjects with higher number of elements that define MetS 68. In addition, oxidative stress is also a critical activator for inflammation. The augmentation in ROS production, NADPH oxidase expression and decrease in antioxidant levels exert significant disturbance in the production of adiponectin, interleukin-6 (IL-6) and monocyte chemoattractant protein-1 (MCP-1) 69. Another postulation is that the link between MetS and inflammation is mediated through the activation of Toll-like receptor (TLR) signalling cascade 70. The increase in exogenous pathogen-associated molecular patterns and endogenous damage-associated molecular patterns during MetS is recognised by TLRs. Subsequently, the binding of these molecular patterns to TLRs activates downstream signalling components to induce the release of inflammatory cytokines 71,72.

Vitamin C showed beneficial effects in alleviating inflammatory response *in vivo* (Table 1). The level of C-reactive protein (CRP) was higher in alloxan monohydrate-induced diabetic rabbits than those supplemented with 150 mg/kg ascorbic acid 25. Comparably, human studies indicated that vitamin C decreased inflammation in MetS conditions (Table 2). In the United States population, vitamin C concentration correlate negatively with the number of MetS indicators and CRP level 42. It was also estimated that the level of IL-6 was increased in Brazilian adults with MetS 43. For MetS patients provided with a balanced diet and 500 mL/day orange juice, higher vitamin C intake but lower CRP and high sensitivity C-reactive protein (hsCRP) levels were observed after three months of intervention 53.

#### Other potential biological functions of vitamin C

Vitamin C has many biological functions aside from its anti-oxidative and anti-inflammatory properties. Vitamin C acts as a co-factor for biosynthesis of carnitine, a molecule that is required in the mitochondrial oxidation of fatty acid 73,74. An increase in the concentration of vitamin C elevates the body's capability to oxidize fat, thus suggesting an inverse relationship between vitamin C status and adiposity 75. Vitamin C also influences the activation of glycolysis via hypoxic signalling. Hypoxia is an event that commonly occurs in MetS-associated conditions due to the uncoupling of oxidation and phosphorylation in mitochondrial respiration and increased oxygen consumption 76. Hypoxic condition favours higher rate of glycolysis through stabilisation of hypoxia-inducible factor-1 (HIF-1) 77. Interestingly, the rate of re-esterification of free fatty acid is directly proportional to the production of glycerol-3-phosphate via glycolysis, resulting in formation and accumulation of TG. High concentration of vitamin C has been reported to degrade HIF-1 and subsequently inhibit glycolysis 78.

In short, oxidative stress and inflammation are two interrelated conditions that characterize the pathophysiology of MetS and its related manifestations. The rise in inflammatory mediators could be responsible for the increase in ROS and vice versa. The anti-oxidative and anti-inflammatory properties of vitamin C are likely the mechanisms of action that substantially reverse MetS. Moreover, fatty acid metabolism and glycolysis can also be affected by the alteration of vitamin C availability and intake.

### Research gap and future perspectives

The current global Recommended Daily Allowance (RDA) for vitamin C varies dramatically across countries, ranges from 40 mg/day in United Kingdom and India to 110 mg/day in European countries to achieve the adequate needs to maintain the balance between oxidative stress and antioxidant protection 79. These values need to be increased for smokers as cigarette smoking increases oxidative stress and metabolic turnover of vitamin C as well as pregnant and lactating women due to the needs of the developing foetus and growing infant 79. Obesity also affects the requirement for vitamin C due to a body weight-dependent dilution effect. Epidermiological studies have demonstrated that increased body weight, fat-free mass or BMI had a negative impact on plasma vitamin C concentration 80-82. Higher body weight might decrease the response towards vitamin C supplementation thus suggesting higher requirement 79. The upper tolerable level of consumption of 2 g/day is set by some countries to avoid osmotic diarrhoea and gastrointestinal disturbance due to excessive ingestion of vitamin C 83. In the context of vitamin C supplementation, it seems reasonable as the doses in the human clinical trials discussed above were ranged between 120 - 500 mg/day. For animals, researchers treated the rats and rabbits with 100 and 150 mg/kg whereby the human equivalent doses based on body surface area were 1.32 g and 3.41 g of vitamin C daily for a 70 kg individual. The dose assigned to the rabbits exceeded the upper tolerable level of consumption for vitamin C, which may require careful consideration for potential side effects. It should also be noted that rodents, unlike humans, can synthesize vitamin C *in vivo* through L-gulonolactone oxidase 84, hence their vitamin C requirements could not be translated to human's directly. This biological difference should be considered carefully when extrapolating the results from animal studies. Besides, most antioxidants do not act alone to exert their protective actions. The effects of vitamin C in protecting MetS should be intepretated together with other dietary antioxidants in the body. In term of absorption, humans absorb vitamin C via the sodium-dependent vitamin C transporter (SVCT1) in the intestine. The capacity of this transporter limits the concentration of vitamin C that can be achieved with oral supplement. Pharmacokinetic study has indicated that oral administration of vitamin C produced a tightly controlled peak plasma concentration 85.

We pointed out several research opportunities in this review. First, the direct effect of vitamin C alone on MetS needs to be confirmed in animals and human populations, which is currently not widely investigated. Second, the combination of vitamin C with other antioxidants as intervention may be advantageous in managing MetS. These compounds may act together against the development of inflammation, oxidative stress and MetS. Third, measurement of the endogenous antioxidants is of interest in the preclinical experimental setting of MetS. The effects of vitamin C on oxidative markers (such as the levels of MDA, ROS and DNA damage) were measured, but the levels of enzymatic and non-enzymatic antioxidants (such as SOD, GPx, CAT and GSH) were not assessed. Vitamin C may protect the endogenous antioxidant defence system from being overwhelmed by oxidative stress, but this speculation needs to be verified. Fourth, a series of inflammation-related biomarkers including leptin, adiponectin, tumour necrosis factor-alpha (TNF-α), interleukin-10 (IL-10) and MCP-1 should be evaluated before and after vitamin C supplementation. These biomarkers can be developed as a panel for early detection of MetS. Fifth, the characterization of the molecular mechanisms underlying the anti-oxidative and anti-inflammatory properties of vitamin C in reversing MetS conditions is warranted. It may be more efficient to unravel and target the signalling pathways orchestrating the generation of ROS and inflammatory cytokines rather than neutralizing them. Even though quenching the oxidative stress and inflammation might be the principal mechanisms of action of vitamin C, other mechanisms that affect the pathophysiology of MetS may be operating as well.

## Conclusion

In conclusion, the promising effects of vitamin C as a dietary supplement to manage MetS and its associated conditions are evident. Individuals with MetS are encouraged to consume adequate vitamin C either from healthy food sources rich in vitamin C or through the use of vitamin C supplements if they fail to achieve the recommended dietary allowance through their daily food intake. Apart from that, vitamin C may complement physical activities, phytochemicals or pharmacological drugs to maximise the therapeutic effects and potentially minimise the side effects of conventional medications.

## Figures and Tables

**Table 1:** Summary on the effects of vitamin C on MetS in animals.

Animal model	Intervention and route of administration	Treatment duration	MetS components				Mechanism of action	Reference
			Obesity	Hyperglycaemia	Hypertension	Dyslipidaemia		
Wistar rats fed on high salt (8% NaCl) diet	Vitamin C (100 mg/kg) - oral	4 weeks	Weight gain: ↓	Glucose: ↓, Insulin: ↓, HOMA-IR: ↓	Systolic BP: ↓	TC: ↓, TG: ↓, LDL-C: ↓, VLDL-C: ↓, AI: ↓	Vitamin C: ↑, Total antioxidant status: ↑, MDA: ↓	22
	Vitamin A (6 mg/kg) + vitamin C (100 mg/kg) + vitamin E (60 mg/kg) - oral		Weight gain: ↓	Glucose: ↓, Insulin: ↓, HOMA-IR: ↓	Systolic BP: ↓	TC: ↓, TG: ↓, LDL-C: ↓, VLDL-C: ↓, AI: ↓, HDL-C: ↑,		
Male and female mutant Wrn*^∆hel/∆hel^* mice	0.4% sodium ascorbate (w/v) - oral	9 months	Visceral fat weight: ↓	FBG: ↓, HOMA-IR: ↓	-	TG: ↓	ROS: ↓, DNA damage: ↓	24
Male albino rabbits induced by alloxan monohydrate	Vitamin C (150 mg/kg) - oral	2 weeks	-	Glucose: ↓	BP: ↓	TG: ↓, HDL-C: ↑, Time for sdLDL oxidation: ↑, PON-1: ↑	Lipid peroxidation: ↓, MDA: ↓, CRP: ↓	25
Male Sprague-Dawley rats	Antioxidants cocktail [containing *S*-adenosylmethionine (0.5 g/kg diet), vitamin C (12.5 g/kg diet) and vitamin E (1.5 g/kg diet)] - oral	43 weeks	Fat pad mass: ↓, Perienteric & epididymal fat mass: ↓	Postprandial glucose: ↔, Postprandial insulin: ↔, RIST: ↑, HISS: ↑	MAP: ↔	-	-	26
Male Sprague-Dawley rats fed with 5% sucrose-supplemented water	Antioxidants cocktail [containing *S*-adenosylmethionine (0.5 g/kg diet), vitamin C (12.5 g/kg diet) and vitamin E (1.5 g/kg diet)] - oral	43 weeks	Whole body fat mass: ↓, Total visceral fat mass: ↓, Perinephric fat mass: ↓, Perienteric fat mass: ↓	Fasting glucose: ↓, Fed glucose: ↓, Fasting insulin: ↓, Fed insulin: ↓	-	TG: ↓, HDL-C: ↑	-	27
Male Sprague-Dawley rats fed with high-fat diet	Fixed dose combination of natural antioxidants (vitamin C, green tea polyphenols and grape seed extract proanthocyanidin) - oral	4 weeks	Body weight: ↓, Average fat coefficient: ↓, Amount of fat in epididymal and perirenal white adipose tissue: ↓, Cell size in adipose tissue: ↓	FBG: ↓, RBG: ↓, PBG: ↓, Glucose tolerance: ↑	-	TG: ↓, LDL-C: ↓, HDL-C: ↑	MDA: ↓	28
Female T2DM KK-ay mice fed with high-fat diet			-	FBG: ↓, RBG: ↓, PBG: ↓, Glucose tolerance: ↑	-	TC: ↓, TG: ↓, LDL-C: ↓, HDL-C: ↑	-	

**Abbreviation:** AI: atherogenic index; BP: blood pressure; CRP: C-reactive protein; DNA: deoxyribonucleic acid; HISS: hepatic insulin sensitizing substance; HOMA-IR: homeostatic model assessment of insulin resistance; LDL-C: low-density lipoprotein cholesterol; MAP: mean arterial pressure; MDA: malondialdehyde; PBG: postprandial blood glucose; PON-1: paraoxonase-1; RBG: random blood glucose; RIST: rapid insulin sensitivity test; ROS: reactive oxygen species; sdLDL: small dense low-density lipoprotein; TC: total cholesterol; TG: triglycerides; VLDL-C: very low-density lipoprotein cholesterol.

**Table 2:** Summary on the effects of vitamin C on MetS in humans.

Study design	Study population	Vitamin C intake / concentration	Findings	Reference
Cross-sectional study	Adults participating in KNHANES 2007 - 2012 (n=27,656; aged ≥20 years)	Intake of vitamin C	MetS subjects had lower vitamin C intake. Higher vitamin C intake lowered the risk of MetS.	29
Cross-sectional study	Adults participating in KNHANES 2008 - 2012 (n=22,671; aged ≥20 years)	Intake of vitamin C	Individuals with high vitamin C intake alone, high physical activity alone as well as both high physical activity and vitamin C intake had lower waist circumference. Individuals with both high physical activity and vitamin C intake had lower TG and higher HDL-C. High vitamin C intake alone, high physical activity alone and both high physical activity and vitamin C intake were associated with low risk of MetS.	30
Cross-sectional study	Adults participating in KNHANES 2013 - 2016 (n=10,351; aged 19 - 64 years)	Intake of vitamin C	Men in the highest tertile of vitamin C intake had a lower prevalence of MetS than those in the lowest tertiles. Women in the highest tertile of vitamin C intake had lower TG than those in the lowest tertile.	31
Cross-sectional study	MetS patients (n=221; aged 54.2 ± 5.73 years) and control subjects (n=329; aged 53.3 ± 5.83 years)	Intake of vitamin C	MetS patients had lower vitamin C intake, SOD activity, β-carotene level but higher MDA content Dietary vitamin C was positively related with serum antioxidant level.	32
Cross-sectional study	Patients diagnosed with colorectal cancer with (n=49; 52.5 ± 13.0 years) and without MetS (n=94; aged 58.0 ± 9.3 years)	Intake of vitamin C	MetS subjects had lower consumption of vitamin C. Higher vitamin C intake lowered the risk of MetS.	33
Cross-sectional study	Volunteers attending Xiangya Hospital Health Management Centre from October 2013 until January 2014 (n=2,069; aged 18 - 84 years)	Intake of vitamin C	Vitamin C intake was inversely associated with MetS. Vitamin C intake showed a negative correlation with waist circumference.	34
Cross-sectional study	Adult Saudis (n=185; aged 19 - 60 years)	Intake of vitamin C	Lower intake of vitamin C caused an increased risk of having MetS.	35
Cross-sectional study	Caucasian healthy young subjects (n=153; aged 20.8 ± 2.7 years)	Intake of vitamin C	Vitamin C intake was positively associated with total antioxidant capacity. Total antioxidant capacity was negatively associated with body weight, waist circumference, waist-to-hip ratio, systolic BP, serum glucose and serum free fatty acids.	60
Case-control study	Healthy women (n=90; aged 41 - 65 years) and MetS women (n=184; aged 45 - 68 years)	Intake of vitamin C	Daily food rations (DFR) showed that the optimal level of 90 - 110% according to standards was only achieved in 8.88% of women with MetS for vitamin C, which was significantly less than the control group.	36
Cross-sectional study	Adults participating in NHANES 2013 - 2016 (n=10,112; aged >19 years)	Intake of 100% fruit juice	100% fruit juice consumers had a higher intake of vitamin C compared to non-consumers. Intake of vitamin C increased with increasing 100% fruit juice consumption level. 100% fruit juice consumers had lower BMI, body weight, waist circumference and HbA1c compared to non-consumers. 100% fruit juice consumers had a lower risk of obesity, elevated waist circumference and MetS as compared to non-consumers.	37
Case-control study	Healthy subjects (n=91; aged 41 - 65 years) and MetS patients (n=182, aged 30 - 65 years)	Intake of vitamin C and plasma vitamin C concentration	Plasma vitamin C concentration was lower in patients with MetS than in healthy subjects. Plasma vitamin C deficiency was more often in MetS patients than in the control group. High concentration of vitamin C was associated with low systolic BP, diastolic BP and HDL-C in MetS patients. No correlation was found between vitamin C intake from the diet and their plasma concentration in MetS patients.	38
Cross-sectional study	Adults participating in NHANES 1988 - 1994 (n=8,808; aged ≥20 years)	Intake of vitamin C and serum vitamin C concentration	Serum vitamin C concentration was lower in MetS subjects than non-MetS subjects. Serum vitamin C concentration was inversely associated with waist circumference, hyperglycaemia and the number of MetS criteria. No difference in dietary intake of vitamin C among participants with and without MetS.	39
Cross-sectional study	Adolescents participating in NHANES 2001 - 2006 (n=4,285; aged 12 - 19 years)	Serum vitamin C concentration	Serum vitamin C concentration was lower in MetS subjects than non-MetS subjects. Serum vitamin C concentration was inversely related to MetS outcomes.	40
Cross-sectional study	Adults participating in NHANES 2001 - 2006 (n=1,574; aged 20 - 85 years)	Serum vitamin C concentration	Serum vitamin C concentration was lower in MetS subjects than non-MetS subjects. Increase in vitamin C was associated with lower odds of MetS.	41
Cross-sectional study	Adults participating in NHANES 2005 - 2006 with MetS (n=2,049; aged ≥20 years)	Serum vitamin C concentration	Vitamin C decreased as BMI and number of MetS components increased. CRP and GGT increased when the number of MetS increased. Having lower than the clinical reference range for vitamin C was associated with significantly higher odds of MetS.	42
Cross-sectional study	Brazilian adults with and without MetS (n=85; aged 22 - 85 years)	Serum vitamin C concentration	Subjects with MetS presented a reduction in serum vitamin C concentration compared to those without MetS.	43
Case-control study	Healthy subjects (n=100; aged 22 - 78 years) and MetS patients (n=100, aged 21 - 73 years)	Plasma vitamin C concentration	Plasma vitamin C concentration of MetS subjects was lower than that of controls.	44,45
Case-control study	Healthy subjects (n=98; aged 41 - 65 years) and MetS patients (n=191, aged 30 - 65 years)	Plasma vitamin C concentration	Plasma vitamin C concentration was lower in patients with MetS than in healthy subjects.	46
Cross-sectional study	Participants with and without MetS from B.P. Koirala Institute of Health Sciences (n=118)	Plasma vitamin C concentration	No difference was observed in plasma vitamin C concentration between non-MetS and MetS participants.	47
Cross-sectional study	Healthy Chinese women, PCOS women and PCOS women with MetS (n=205; aged 21 - 40 years)	Serum vitamin C concentration	No difference was detected in serum vitamin C concentration between PCOS women with and without MetS. Lower SOD, total antioxidant activity and higher MDA were detected in PCOS women with MetS as compared to healthy controls.	48
Randomised double-blind, placebo-controlled trial	Adults participating in SUpplementation en VItamines et Mineraux AntioXydants (SU.VI.MAX) primary prevention trial (n=5,220)	Supplementation with antioxidants (120 mg vitamin C, 30 mg vitamin E, 6 mg β-carotene, 20 mg zinc and 100 μg selenium)	Antioxidants supplementation for 7.5 years did not affect the risk of MetS. Higher baseline serum vitamin C concentration was associated with a lower risk of MetS (OR=0.53; 95% CI 0.35 - 0.80).	49
Randomised controlled trial	MetS patients (n=141; aged 30 - 50 years)	Supplementation with vitamin C (500 mg/day) alone or in combination with physical activity	Taking vitamin C with exercise lowered waist circumference and increased HDL-C compared to placebo. Taking either vitamin C or vitamin C with exercise lowered TG compared to placebo.	50
Randomised double-blinded, placebo-controlled trial	MetS patients (n=96, aged 30 - 60 years)	Supplementation with vitamin C (500 mg/day) alone or in combination with physical activity	Vitamin C plus exercise decreased systolic BP compared to placebo. Vitamin C supplementation decreased BMI and LDL-C compared to the placebo group.	51
Double-blinded, controlled trial	Postmenopausal women with T2DM (n=69; aged 50 - 65 years)	Supplementation with zinc (5 mg) plus vitamin C (300 mg)	Supplementation with zinc plus vitamin C decreased systolic and diastolic BP but increased FBG and HDL-C.	52
Randomised controlled trial	MetS patients (n=72; aged 48 ± 9 years)	Adopted a balanced diet only or adopted a balanced diet plus orange juice (500 mL/day)	Both interventions decreased body weight, BMI, waist circumference, fat mass, visceral fat area, glucose, TC, HDL-C, systolic BP, diastolic BP and increased antioxidant capacity. Only patients adopting a balanced diet plus orange juice had lower insulin, insulin resistance, CRP and hsCRP.	53
Randomised controlled trial	MetS patients (n=81; aged 35 - 65 years)	Low sodium vegetable juice (8 or 16 ounces/day)	Groups consuming vegetable juice had a higher intake of vitamin C, lower leptin level and lost more weight.	54

**Abbreviation:** BMI: body mass index; BP: blood pressure; CRP: C-reactive protein; DFR: daily food rations; FBG: fasting blood glucose; GGT: γ-glutamyl transferase; HbA1c: glycated haemoglobin; HDL-C: high-density lipoprotein cholesterol; hsCRP: high sensitivity C-reactive protein; KNHANES: Korea National Health and Nutrition Examination Survey; MDA: malondialdehyde; MetS: metabolic syndrome; PCOS: polycystic ovary syndrome; SOD: superoxide dismutase; TC: total cholesterol; TG: triglycerides.

## Abbreviations

AI: atherogenic index
apoA1: apolipoprotein A-1
BMI: body mass index
BP: blood pressure
CAT: catalase
CI: confident interval
CRP: C-reactive protein
DFR: daily food rations
DNA: deoxyribonucleic acid
FBG: fasting blood glucose
FRAP: total ferric antioxidant power
GGT: γ-glutamyl transferase
GOT: glutamic-oxaloacetic transaminase
GPT: glutamic-pyruvic transaminase
GPx: glutathione peroxidase
GSH: reduced glutathione
HbA1c: glycated haemoglobin
HDIR: hepatic insulin sensitizing substance (HISS)-dependent insulin resistance
HDL-C: high-density lipoprotein cholesterol
HISS: hepatic insulin sensitizing substance
HOMA-IR: homeostatic model assessment of insulin resistance
hsCRP: high sensitivity C-reactive protein
IL-6: interleukin-6
IL-10: interleukin-10
KNHANES: Korea National Health and Nutrition Examination Survey
LDL-C: low-density lipoprotein cholesterol
MAP: mean arterial pressure
MCP-1: monocyte chemoattractant protein-1
MDA: malondialdehyde
MetS: metabolic syndrome
MUFA: monounsaturated fatty acid
NaCl: sodium chloride
NADPH: nicotinamide adenine dinucleotide phosphate
NF-κB: nuclear factor-kappa B
NHANES: National Health and Nutrition Examination Survey
OR: odd ratio
PBG: postprandial blood glucose
PCOS: polycystic ovary syndrome
PON-1: paraoxonase-1
PUFA: polyunsaturated fatty acid
RBG: random blood glucose
RDA: Recommended Daily Allowance
RIST: rapid insulin sensitivity test
ROS: reactive oxygen species
sdLDL: small dense low-density lipoprotein
SFA: saturated fatty acid
SH: sulfhydryl groups
SOD: superoxide dismutase
T2DM: type 2 diabetes mellitus
TBARS: thiobarbituric acid reactive substances
TC: total cholesterol
TFA: trans-unsaturated fatty acid
TG: triglycerides
TLR: Toll-like receptor
TNF-α: tumour necrosis factor-alpha
VLDL-C: very low-density lipoprotein cholesterol
